# RETRACTION: Clinical Application of Posterior Tibial Artery or Peroneal Artery Perforator Flap in Curing Plate Exposure After Ankle Fracture Fixation

**DOI:** 10.1111/iwj.70599

**Published:** 2025-04-21

## Abstract

**RETRACTION:** QianD. , ZhengJ. , WangK. , LuH. , YuB. , XuM. , QianY. , ZhangJ. , and ShaoS. , “Clinical Application of Posterior Tibial Artery or Peroneal Artery Perforator Flap in Curing Plate Exposure After Ankle Fracture Fixation,” International Wound Journal 21, no. 2 (2024): e14423, 10.1111/iwj.14423.PMC1082871537770196

The above article, published online on 28 September 2023, in Wiley Online Library (http://onlinelibrary.wiley.com/), has been retracted by agreement between the journal Editor in Chief, Professor Keith Harding; and John Wiley & Sons Ltd. Following an investigation by the publisher, all parties have concluded that this article was accepted solely on the basis of a compromised peer review process. The editors have therefore decided to retract the article. The authors did not respond to our notice regarding the retraction.

**RETRACTION:** D. Qian , J. Zheng , K. Wang , H. Lu , B. Yu , M. Xu , Y. Qian , J. Zhang , and S. Shao , “Clinical Application of Posterior Tibial Artery or Peroneal Artery Perforator Flap in Curing Plate Exposure After Ankle Fracture Fixation,” International Wound Journal 21, no. 2 (2024): e14423, 10.1111/iwj.14423.PMC1082871537770196

The above article, published online on 28 September 2023, in Wiley Online Library (http://onlinelibrary.wiley.com/), has been retracted by agreement between the journal Editor in Chief, Professor Keith Harding; and John Wiley & Sons Ltd. Following an investigation by the publisher, all parties have concluded that this article was accepted solely on the basis of a compromised peer review process. The editors have therefore decided to retract the article. The authors did not respond to our notice regarding the retraction.

